# Formative psychosocial evaluation using dynamic networks: trauma, stressors, and distress among Darfur refugees living in Chad

**DOI:** 10.1186/s13031-019-0212-2

**Published:** 2019-06-26

**Authors:** Candace Mootoo, Christine Fountain, Andrew Rasmussen

**Affiliations:** 1 Fordham University, 441 East Fordham Road, Dealy 226, Bronx, NY 10458 USA; 2 Fordham University at Lincoln Center, 113 West 60th Street, LL 917B, New York, NY 10023 USA

**Keywords:** Displacement stressors, Traumatic events, Impairment, Symptoms, Dynamic networks, Refugee populations

## Abstract

**Background:**

Research on the impact of various types of stressors on refugee wellbeing may not readily inform those designing interventions about the supports that will be most helpful in particular settings. Composite variables used in psychosocial research that represent overarching types of stressors provide only vague information about intervention targets. Dynamic networks model individual phenomena separately (i.e., specific stressors and symptoms of distress) to inform how phenomena interact with each other in ways that may be useful for individuals planning interventions in humanitarian aid settings.

**Methods:**

Using archival data from Darfur refugees, we used a dynamic networks approach to model relationships between locally-validated measures of traumatic events, displacement stressors, impairment, and distress.

**Results:**

Findings aligned with previous research on the centrality of basic needs in refugee populations. Further, specific stressors were highlighted as particularly impactful for this population, and sleep and physical difficulties emerged as key aspects of distress and impairment, suggesting areas for targeted intervention. *Conclusions*: Dynamic network approaches may be fruitful for identifying setting-specific intervention targets and maximizing the impact of limited resources in humanitarian aid settings.

**Electronic supplementary material:**

The online version of this article (10.1186/s13031-019-0212-2) contains supplementary material, which is available to authorized users.

## Background

The past two decades have witnessed significant advances in research on how exposure to war trauma interacts with multiple stressors involved in humanitarian crises to impact refugees’ psychosocial wellbeing. Guidelines for mental health and psychosocial support services (MHPSS) (e.g., Inter-Agency Standing Committee (IASC) [15] and psychosocial theories [3, 4, 22, 24, 25, 33] both acknowledge that stressors at multiple levels are determinants of psychosocial distress. Several research programs have combined advanced statistical modeling with sensitivity to local belief systems and disaster contexts (e.g., [38]), and in general, evince transactional relationships between stressors across social ecological levels that impact refugees’ wellbeing. However, several of the conventions of such research present barriers to applying findings to services. For example, it is common for researchers to reduce complicated measurement models to conceptual equations that collapse diverse stressors (e.g., war trauma and daily stressors, [22]) or diverse indicators of psychosocial distress (i.e., symptoms; e.g., [34, 36]) into broad constructs. This type of categorization is the norm in psychological research, but it may not be particularly illuminating to those tasked with targeting individual problems in specific humanitarian aid settings. For example, when researchers contend that the effect of war trauma events on psychosocial wellbeing is mediated by daily stressors across humanitarian aid crises [17, 29], this provides only vague notions to MHPSS providers about which daily stressors to target for intervention within a given crisis. Advances in psychosocial research have been critical in supporting multilevel, multifaceted interventions, but have provided less in terms of answering the practical question, “But what are the stressors causing so much distress in *this* crisis?”

To a certain extent, the tension between researchers and MHPSS providers reflects the inevitable tension between reliability and validity. Researchers wish to establish theory across populations, while practitioners wish to find what works for the individuals or groups with whom they work. Some of this tension may be due to methodological conventions used in formative evaluation research—specifically, the use of latent and non-latent composite variables. We propose that avoiding composite variables and treating stressors and symptoms as individual phenomena within dynamic networks may provide practitioners with a more practicable approach. This is consistent with criticisms that emphasize the futility of using composite variables to elucidate how individual phenomena interact with and have causal impacts on each other [32].

Consider two points related to the problematic nature of latent and non-latent composite variables: (1) Such variables are ubiquitous and often used in planning MHPSS. Most psychosocial research relies on sum scores of stressors (e.g., trauma event checklists; [27]) and total scores from psychological measures, which assume that individual disorders are discrete, latent variables (e.g., questionnaires, clinical interviews, and many tools that measure cultural constructs of distress (CCDs); see [18] for a review of such measures). (2) Composite variables are often too broad to guide MHPSS practitioners in targeting the stressors or symptoms that are most problematic. With composite variables, specific phenomena of interest represent only pieces of larger constructs. For example, responses to a questionnaire item concerning problems getting water in a refugee camp are of interest only in as far as they contribute to a composite score for lack of basic needs [29]. However, this composite score does not give practitioners a sense of the relative importance of this issue compared with other basic needs, what getting water is associated with, or factors that may predict or result from limited access to water, etc. Similarly, a total score on a depression measure validated for the local cultural context may be useful for screening individuals into more intense services, but it does not suggest which symptoms are especially problematic and should be prioritized in intervention. There may be limitations to latent variable approaches that composite scores avoid (e.g., latent variable modeling often results in excluding symptoms that are not correlated with the construct without regard for their impact on distress; [19]), but both latent and non-latent variable approaches obscure elements that are of interest on their own – one of the main culprits in the tension between research and implementing MHPSS interventions.

Dynamic network analysis provides a methodology that models individual phenomena of interest separately. Clinical applications of network theory have responded to the limitations of latent variable modeling by representing visualizations of causal networks of symptoms [1, 5, 21]. In dynamic network models, each element (stressor or symptom) is a node, and ties between nodes represent covariance between elements. Association networks are based on first-order (i.e., Pearson) correlations between nodes, and concentration networks on partial correlations [5]. Partial correlations account for background associations between elements in association networks, which can identify potential causal pathways between elements. The importance of each node within the network is observed by calculating its centrality. Centrality can be measured in a number of ways related to the number, strength, and pattern of ties associated with each node. Practically speaking, if a respondent endorses a node that is particularly central to a network, then the probability of that individual endorsing other nodes is greater than if the individual endorses a peripheral node [13]. Visualizing networks maps the associations between all elements simultaneously, allowing researchers to note relative placements of nodes that indicate their centrality or lack thereof. The past half-decade has witnessed multiple developments in dynamic network approaches that have improved its rigor, including measures of expected influence centrality, which consider the direction of correlation effects and conceptualize that nodes with many negative ties, for instance, are particularly influential in a network [30] .

In the psychosocial humanitarian aid literature, two studies have used dynamic networks to conceptualize and visualize trauma events, displacement stressors, and distress as nodes in a network of problems [7, 16]. Working with the standard conventions established for dynamic network analyses, these studies presented association and concentration networks of elements in northern Ugandan and Sri Lankan internally displaced persons camps (respectively). De Schryver et al. [7] revealed that although depression and PTSD symptoms clustered closely together in one subnetwork and stressful wartime events and daily stressors clustered closely in another, there were several important intermediary nodes connecting the two subnetworks. In addition, there was considerable variety in centrality—the degree to which individual elements connect to others within networks—with traumatic wartime events and daily stressors having greater centrality on average than symptoms. Jayawickreme et al. [16] employed a locally developed measure of trauma exposure, stressful life problems, and symptoms of psychopathology and found that social problems in particular were the most central class of elements within the network.

The current study presents a dynamic network analysis of archival data from Darfur refugees that have previously been examined using a composite variable approach and were collected in 2007 and 2008 [28, 29]). These data used locally developed measures of distress—*hozun* and *majnun*, Darfur-relevant CCDs—locally developed measures of stressors, both traumatic and otherwise, and measures of functional impairment. Findings from these studies were that basic needs and perceived safety were more highly associated with distress than the number of traumatic events, that these stressors mediated the effect of traumatic events on subsequent distress [29]; and that Darfur CCDs were comprised of intrusion and depressed affect, psychotic reactions, and despair, and predicted functional impairment better than PTSD scores [28]. In comparing dynamic network results to these findings, we wish to introduce dynamic networks as a MHPSS formative analysis tool and note how such an approach might be used to identify relevant stressors and symptoms in other settings.

## Methods

### Participants

The sample was composed of 863 Darfur refugees residing in two refugee camps in Eastern Chad. Participants were retained by identifying a random sample of 7.5% of the adult population from both camps, based on United Nations High Commissioner for Refugees (UNHCR) Registration records. Ethnic group of origin was largely Masalit (*n* = 834, 96.6%), and all participants were Sunni Muslim. The sample was nearly two-thirds female (*n* = 561, 65%), which mirrored the gender representation in the camps. The average age of participants was 33.8 (SD = 14.40). Trained Sudanese interviewers conducted structured interviews in Masalit (*n* = 600, 69.5%), Arabic (*n* = 227, 26.3%) or another local language (*n* = 3, .3%; some were missing this information, *n* = 33, 3.8%). More detailed information about the sample and data collection procedures is published elsewhere [28].

### Measures

#### Hozun

This measure of psychological distress, which translates to “deep sadness”, was developed in previous work [28] that identified key symptoms of distress in this population by holding focus groups and card sorts with traditional healers. It was validated by establishing internal reliability with the current sample (α = .88) and confirming concurrent validity by finding positive correlations with other validated measures of distress, including BSI ratings of depression. It is an 18 item measure of psychological distress (e.g., feeling hopeless about the future, irritability or outbursts of anger, flashbacks, feeling lonely, difficulty falling asleep, lack of appetite, thinking too much, feeling bad about surviving) which shares many symptom features of the Diagnostic and Statistical Manual – 5 (DSM-5; APA, 2013) constructs of Major Depressive Disorder (MDD) and intrusion and distress from Posttraumatic Stress Disorder (PTSD). Participants rated how distressing each of these experiences was on a scale of 0 to 4 (0 = *not at all distressing*, 4 = *extremely distressing*).

#### Potentially traumatic events

Potentially traumatic events (PTEs) that refugees experienced during attacks were also identified and validated in previous work [28], yielding 13 potentially traumatic events (e.g. being beaten, being chased). Participants were asked whether they had experienced each event. Each personally experienced event received a score of one, with items that were not endorsed coded zero.

#### Displacement stressors

Displacement stressors were identified in previous work [28] and included eight items representing basic needs (e.g., getting food in the camp) and eight items pertaining to safety concerns (e.g., not feeling safe in the camp). Participants rated how stressful each of these experiences was on a scale of 0 to 2 (0 = *not stressful*, 2 = *very stressful*). Regarding basic needs, the item pertaining to difficulty accessing money was not used in these analyses, given the poor variability of this variable (i.e., nearly all participants endorsed stress due to this experience).

#### Functional impairment

The World Health Organization’s Disability Assessment Schedule-Version II (WHODAS-II; [39]) is a measure of functional impairment that has been used extensively with international war-affected populations (e.g., [23]). It consists of 12 items measuring past-month functioning across the domains of mobility, self-care, life activities, cognition, interpersonal interactions, and community participation (e.g., “In the past 30 days, how much of a problem did you have in joining in community activities in the same way as anyone else can?”) and 1 item asking about overall impairment (i.e., “Overall, how much did these difficulties interfere with your life?”). Responses fall on a 0 to 4 scale (0 =* no difficulty*; 4 = *extreme difficulty or cannot do*). For this study, the measure was translated from English into Arabic and back-translated into English by translators who were blind to the content of the original English version. For the response scale to be understood by participants, they were given a visual scale, an approach that has been used by other researchers in international contexts (e.g., [2]). The scale was represented by either a man or woman, depending on participant gender, carrying increasing amounts of wood (0 = *figure carrying a few sticks of wood without difficulty*; 4 = *figure managing many sticks of wood with great difficulty*). Internal reliability of this measure in the current study was appropriate (α = .87).

### Translation and interview procedures

As Arabic is the primary written language in the region that is used in formal education, all measures were translated into and written in Arabic. Interviews were either conducted in Arabic or Masalit, depending on the primary language of the participant; for Masalit-language interviews, trained interviewers and interpreters translated into Masalit during the interview. To confirm consistency of the translations, interviewers and interpreters held regular meetings to confirm the vocabulary used during translation. This approach has been taken with other psychological research in refugee populations that use nonwritten languages (e.g., [31]).

### Data analysis

#### Association network

**Fig. 1:**
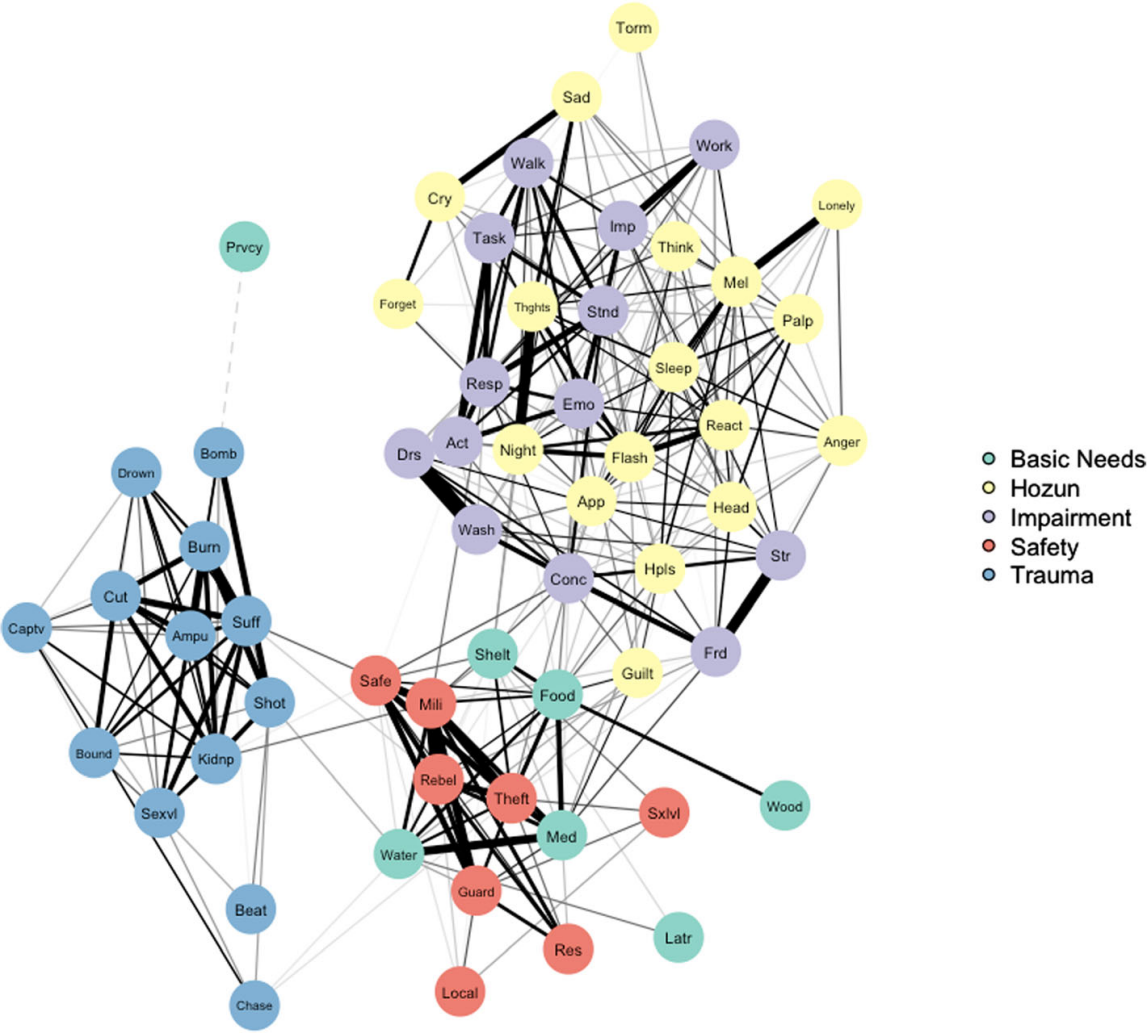
Association network with edges indicating zero-order correlations greater than *r*= |0.30| between nodes. Thickness of edges corresponds to correlation magnitude, and dashed edges indicate negative correlations

In association networks, edges represent zero-order correlations between nodes. In this study, nodes represent potentially traumatic events, displacement stressors (both basic needs and perceptions of safety), functional impairment, and symptoms of distress, and the edges represent the zero-order correlations between them (Fig. 1). To prepare the network visualization, we utilized the R package *qgraph* [9] and a matrix of zero-order Pearson correlations between all nodes in the network. The Fruchterman and Reingold [14] algorithm situates nodes that are more strongly correlated with each other closer to the center of the network. As done in previous network studies of psychopathology (e.g., [21]), correlations with effect size less than *r* = |0.30| were not represented in the visualization.

#### Concentration network

In concentration networks, edges represent partial correlations, or correlations between nodes that statistically control for all other associations in the network. These networks can identify potential causal pathways [11], but given that they often include minute correlations that are not likely to correspond to causal relationships, we employed the graphical least absolute shrinkage and selection operator (GLASSO) procedure in *qgraph* [37]. This approach reduces very small edges to zero in partial correlation networks, rendering them more interpretable [8], and has been increasingly used in studies modeling symptoms of distress (e.g., [6, 20]). This approach detects ordinal and continuous variables and calculates Pearson, polychoric, and polyserial correlations as appropriate. The EBIC hyperparameter was set to 0.5, the recommended approach for creating a network that possesses greater parsimony and specificity [12]. Again, the Fruchterman and Reingold [14] algorithm situates nodes that are more strongly correlated with each other closer to the center of the network. In order to evaluate network accuracy and stability, we used the R package *bootnet* [10] based on the approaches described by Epskamp et al. [8]. To evaluate network edge accuracy, we utilized bootstrapping techniques to calculate the 95% confidence intervals of network edges. To evaluate centrality index stability, we compared centrality values from the original sample to values from increasingly smaller bootstrapped subsamples, yielding the greatest proportion of cases that may be dropped from the sample while maintaining a correlation of *r* greater than or equal to 0.70 between original and bootstrapped values.

#### Node centrality

For both association and concentration networks, we used the R package *qgraph* to calculate four types of centrality for each node: closeness, strength, betweenness, and expected influence. Closeness is a measure of the inverse of the mean shortest distance between a node and all other nodes that indicates the proximity of a node to all others in the network. Strength is a measure of the sum of correlations between a node and all adjacent nodes that indicates the number and magnitude of edges connected to a node. Betweenness is a measure of how often a node falls on the shortest path between pairs of nodes that indicates the extent to which a given node connects other nodes in the network. Expected influence is a measure of potential to impact other nodes in the network, as it considers both the direction and magnitude of correlations between nodes, with greater values assigned to individual nodes that possess either many positive or many negative correlations with other nodes in the network. To facilitate interpretation, centrality values were normalized on a scale of 0 to 1.

## Results

### Descriptives

#### PTE exposure and displacement stressors

Table 1 includes the percentage of participants who experienced PTEs. The most common PTEs were being chased (58.2%), beaten (44.0%), bombed (37.8%), or shot (31.5%). Table 1 also includes the percentage of participants who endorsed that displacement stressors were “very stressful”. The most common stressors related to basic needs were getting firewood (72.9%), finding shelter (64.9%), and accessing latrines (63.4%). Common stressors related to safety concerns were problems with locals near camp (61.1%) and sexual assault in or near the camp (56.4%).

**Table 1:** Exposure to PTE’s and endorsement of displacement stressors

*PTEs*	Endorsed (%)	*Displacement Stressors (Basic Needs)*	Very Stressful (%)
Being beaten	44.0	Getting food	50.1
Being shot	31.5	Getting firewood	72.9
Being burnt	14.3	Finding shelter	64.9
Limb amputation	10.9	Finding privacy	46.7
Being bound	14.7	Getting water	44.3
Being stabbed or cut	12.2	Getting medical help	41.7
Suffocation or strangulation	10.9	Accessing latrines	63.4
Bombing	37.8		
Being chased	58.2	*Displacement Stressors (Safety Concerns)*	
Sexual violence	22.7	Problems with other camp residents	10.8
Being drowned	8.7	Problems with locals near camp	61.1
Held captive	27.1	Problems with camp guards	17.8
Kidnapped	15.6	Sexual assault in or near camp	56.4
		Recruitment by rebels in camp	17.7
		Property taken by others	40.1
		Not feeling safe in camp	38.2
		Threats to camp from militant groups	23.6

#### Impairment and distress

Table 2 includes the mean ratings for each type of impairment, with the most strongly endorsed items, on average, including difficulties standing for long periods of time, walking long distances, and overall impact on life. Table 2 also includes the mean ratings for each symptom of distress, with the most strongly endorsed symptoms, on average, including deep sadness, thinking too much, recurrent thoughts, and recurrent nightmares.

**Table 2:** Endorsement of functional impairment and distress

Functional Impairment	M	SD	Distress	M	SD
Standing for long periods	3.16	1.14	Deep sadness	3.29	1.07
Managing household responsibilities	2.61	1.29	Crying uncontrollably	2.86	1.30
Learning a new task	2.89	1.25	Lack of appetite	2.63	1.30
Joining community activities	2.48	1.42	Forgetfulness	2.58	1.36
Emotionally affected by health	2.73	1.25	Thinking too much	3.12	1.06
Concentrating on doing something	2.19	1.39	Palpitations	2.51	1.29
Walking a long distance	3.19	1.12	Headaches	2.51	1.31
Washing whole body	1.56	1.47	Being tormented	2.63	1.35
Getting dressed	1.44	1.44	Feeling bad about surviving	1.67	1.49
Dealing with strangers	2.29	1.43	Recurrent thoughts	3.36	0.95
Maintaining a friendship	2.12	1.49	Recurrent nightmares	3.20	1.02
Day to day work	2.60	1.31	Flashbacks	2.94	1.18
Impact on life	3.15	1.04	Physiological reactivity at cues	2.64	1.26
			Difficulty falling asleep	2.87	1.22
			Irritability or outburst of anger	2.14	1.45
			Feeling lonely	2.62	1.42
			Feeling melancholy	2.88	1.25
			Feeling hopeless about future	2.41	1.53

### Node type

The association network, depicted in Fig. 1, illustrates interrelationships between node types, with nodes corresponding to *hozun* and functional impairment clustering together, as with nodes corresponding to lack of basic needs and perceptions of safety. To aid interpretation of the network visualizations in this paper, node abbreviation descriptions are included in Additional file 1. In contrast, experiences of potentially traumatic events were relatively isolated in the network. This was confirmed by the results of one-way ANOVA permutation tests, which indicated that PTE nodes possessed significantly lower closeness centrality and expected influence than *hozun*, functional impairment, and perception of safety nodes (*p* < .05 for each comparison) as well as significantly lower strength centrality than *hozun* and functional impairment nodes (*p* < .01 for each comparison). There were no other statistically significant results of permutation tests. For the association network, mean centrality values by node type are included in Table 3.

**Table 3:** Descriptive statistics for association network centrality measures by node type

	Betweenness	Closeness	Strength	Expected Influence
Basic Needs	0.138	0.801	0.708	0.653
*Hozun*	0.074	0.822	0.790	0.780
Impairment	0.117	0.860	0.833	0.811
Safety	0.165	0.838	0.776	0.772
Trauma	0.060	0.700	0.637	0.578

**Fig. 2:**
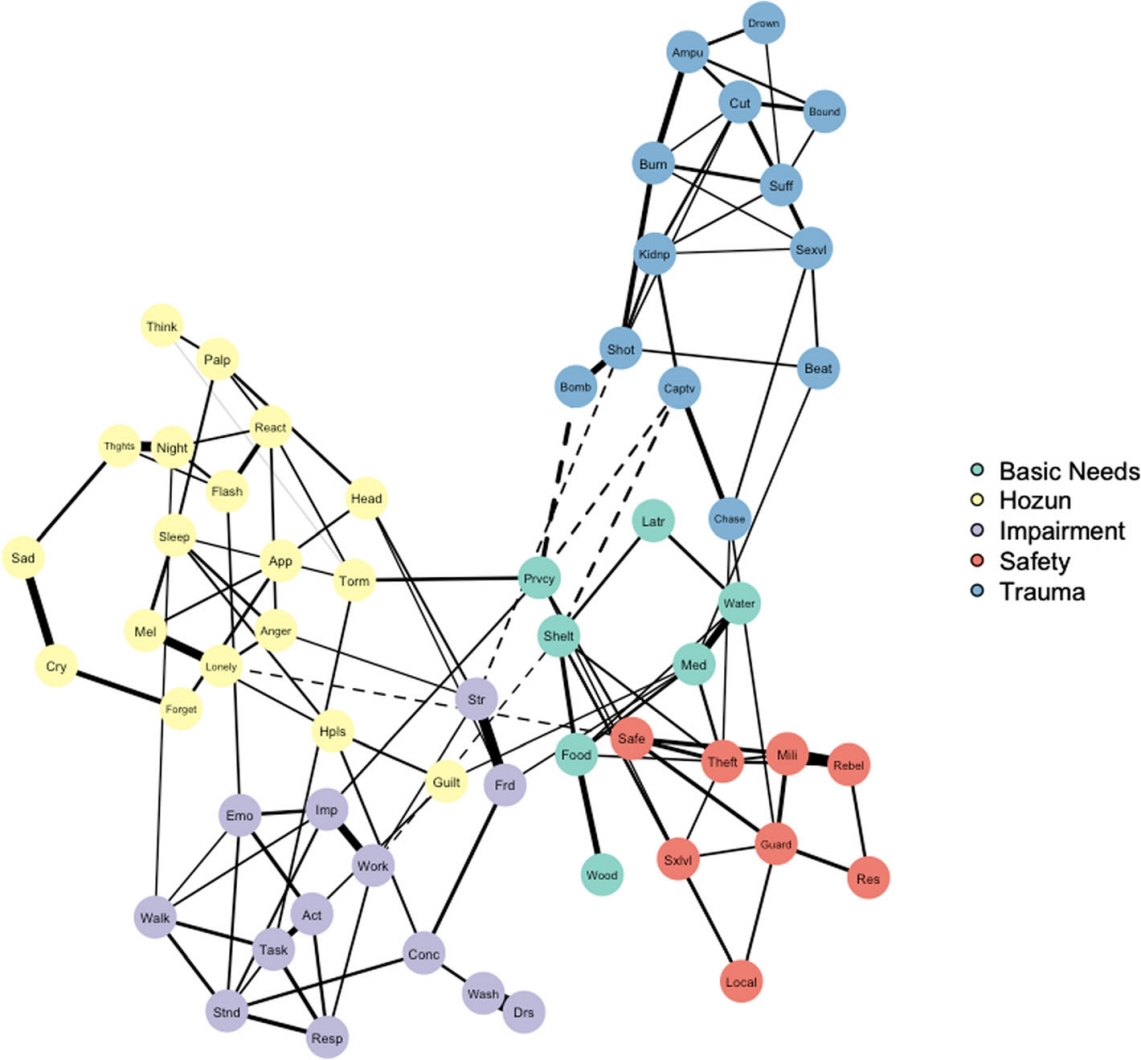
Concentration network, with edge minimum of *r* = |.08|. Edges indicate partial correlations between nodes. Thickness of edges corresponds to correlation magnitude, and dashed edges indicate negative correlations

The concentration network, depicted in Fig. 2, portrays a similar result with regard to the relative isolation of PTEs in the network, though differences in node centrality were not significantly different. Also revealed is the extent to which basic needs nodes fall between and appear to connect PTEs with *hozun* and functional impairment. This is supported in part by the results of one-way ANOVA permutation tests, which indicated that basic needs nodes have significantly greater closeness centrality than *hozun* nodes (*p* < .05). There were no other statistically significant results of permutation tests. For the concentration network, mean centrality values by node type are included in Table 4.

**Table 4:** Descriptive statistics for GLASSO network centrality measures by node type

	Betweenness	Closeness	Strength	Expected Influence
Basic Needs	0.369	0.867	0.710	0.554
*Hozun*	0.165	0.776	0.688	0.665
Impairment	0.244	0.823	0.763	0.701
Safety	0.219	0.825	0.708	0.677
Trauma	0.247	0.769	0.713	0.589

### Concentration network accuracy and stability

The evaluation of network edge accuracy revealed overlapping bootstrapped confidence intervals, suggesting that edge value magnitude should be interpreted with caution. Regarding network stability, average correlations between the centrality values from the original sample and increasingly smaller bootstrapped subsamples were calculated. The correlation stability coefficient—the greatest proportion of cases that can be dropped from the sample while maintaining a correlation between original and bootstrapped centrality values of *r* is equal to .70 or greater—was 0.362 for closeness, 0.284 for strength, and 0.205 for betweenness. Preferably, these coefficients should be above 0.50 but not below 0.25, suggesting that the closeness and strength indices meet minimum stability requirements but that differences in centrality between nodes should not be over-emphasized [1, 8]. As expected influence is a relatively new metric, statistical packages for computing its stability are not yet available. Results of the analyses pertaining to network accuracy and stability are included in Additional files 2 and 3.

### Individual nodes

**Fig. 3:**
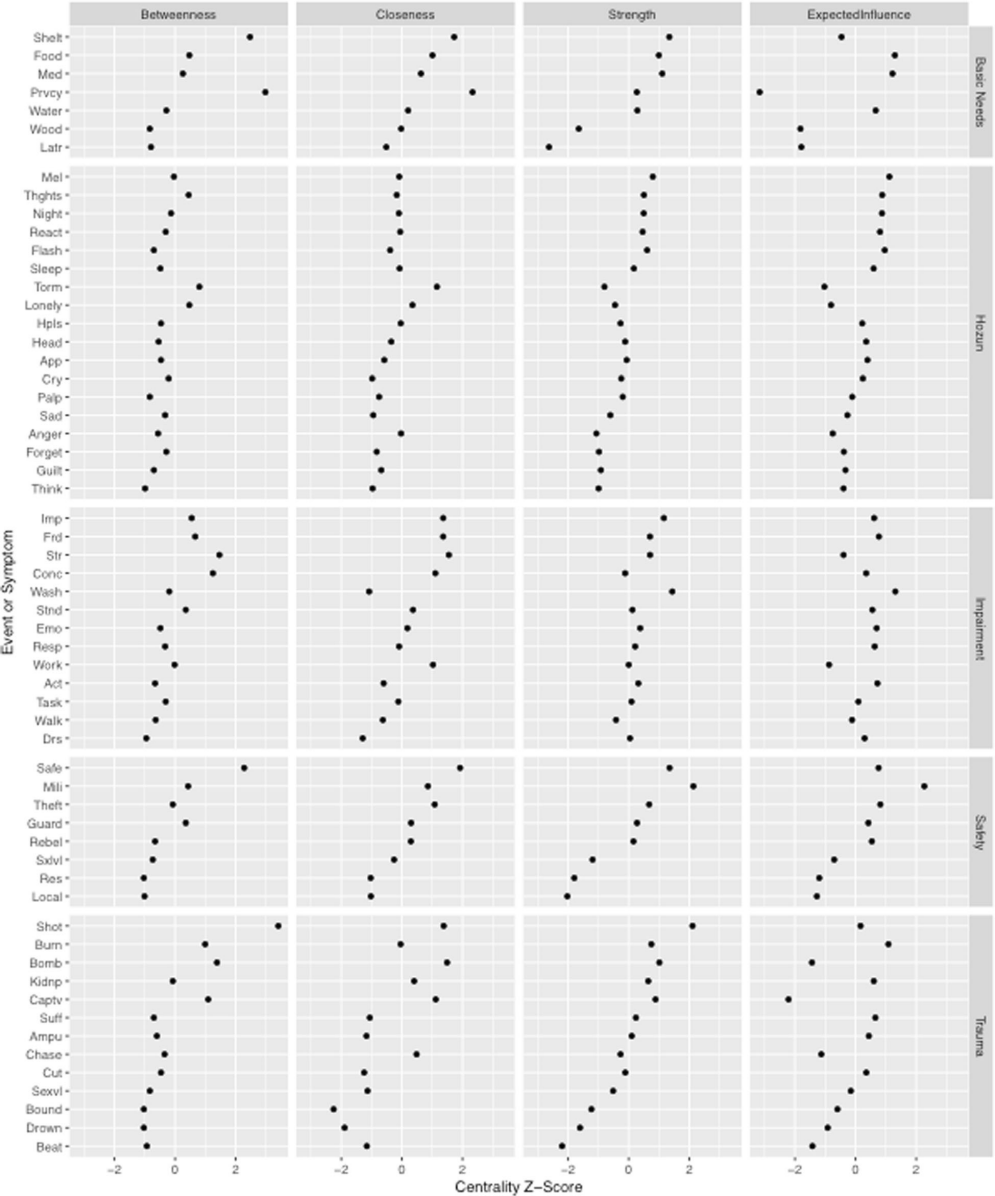
GLASSO network standardized centrality values

Due to the increased interpretability of links between nodes in concentration networks (e.g., [32]), we will focus on these results. The standardized centrality values of individual nodes within concentration networks are depicted in Fig. 3. Given relatively weaker stability of betweeenness centrality indices, results will also focus on closeness and strength centrality indices.

Nodes that possess high closeness values are the most proximal to other nodes in the network and may therefore indicate that other problems are present. Regarding displacement stressors related to basic needs, the nodes with highest closeness values included finding privacy (Prvcy), finding shelter (Shelt), and procuring food (Food); concerning safety concerns, they were not feeling safe in camp (Safe), threats to camp from militant groups (Mili), and property being taken by others (Theft). Concerning PTEs, nodes with highest closeness were being shot (Shot), bombed (Bomb), or held captive (Captv). Among symptoms of distress, nodes with highest closeness were being tormented (Torm) and feeling lonely (Lonely). Finally, among types of impairment, nodes with highest closeness were overall impact on life (Imp), maintaining friendships (Frd), and dealing with people one does not know (Str).

Concerning strength centrality, nodes that are elevated in value are most strongly tied to other nodes in the network and may represent problems that tend to co-occur with others in the network. Regarding displacement stressors related to basic needs, the nodes with highest strength centrality included finding shelter (Shelt), procuring food (Food), and getting medical help (Med); concerning safety concerns, they were the same as the nodes with highest closeness: not feeling safe in camp (Safe), threats to camp from militant groups (Mili), and property being taken by others (Theft). Concerning PTEs, nodes with the highest strength centrality were also the same as the nodes with highest closeness: being shot (Shot), bombed(Bomb), or held captive (Captv). Among symptoms of distress, nodes with highest strength were feeling melancholy (Mel), having flashbacks (Flash), having recurring thoughts (Thghts), having recurrent nightmares (Night), and being physiologically reactive (React). Among types of impairment, nodes with highest strength were washing one’s body (Wash), overall impact on life (Imp), maintaining friendships (Frd), and dealing with people one does not know (Str).

Finally, in terms of expected influence, these nodes are thought to have the greatest potential to impact other nodes in the network. The nodes with highest expected influence largely overlapped with the nodes possessing highest closeness or strength, as described above. The few exceptions were as follows: regarding displacement stressors – procuring water (Water), concerning PTEs – being kidnapped (Kidnp) and suffocated/strangled (Suff), and regarding impairment – being emotionally affected by health problems (Emo) and joining community activities (Act).

## Discussion

Consistent with the findings from research using a composite variable approach with this data [29], basic needs were central within the larger constellation of problems in these camps. This mirrors research findings from other displaced populations using both composite variable analysis and network approaches [16, 17]. Beyond examining larger categories, the network approach treats specific problems independently, and in the current study we found that several specific stressors played key roles. Using Rasmussen et al. [29] findings would lead psychosocial aid workers to address basic needs and safety concerns, which were associated with psychosocial distress; this provides some direction, but could result in targeting any number of issues. Using the current study findings would lead them to focus on specific issues indicated by nodes with both high closeness and strength: shelter, food, safety, theft, threats from militia, and interpersonal challenges like dealing with strangers or maintaining friendships.

Further, interrelationships between nodes in the concentration network enable us to consider particular psychological problems that co-occur, which in turn may suggest specific areas for psychosocial intervention. In particular, sleep difficulties were strongly linked to multiple other distress nodes in the *hozun* network, including anger, feeling melancholy, feeling hopeless about the future, palpitations, and nightmares. The critical role of sleep for mental health and wellbeing is well-documented (e.g., [35]), and network visualizations can help illustrate the dynamic connections between sleep and several other symptoms of psychopathology (e.g., [5]). These relationships are correlational, precluding assumptions of causality; nevertheless, these pathways indicate the potentially central role of sleep with regard to the extent to which it may be affected by or contributing to experiences of distress in this population. Given the convergence of data and theory, it seems reasonable that addressing sleep problems might be an opportune target for intervention. Although all of these symptoms were part of the CCD *hozun* identified by Rasmussen et al. [28], such network findings provide targets for intervention in ways that simply defining the set of symptoms cannot.

The network visualization revealed a cluster of relationships indicating links between physical difficulties and functional impairment. That is, walking for long distances was strongly linked to both standing for long periods and learning new tasks. Standing for long periods was also linked to difficulty taking care of household responsibilities, which was linked to difficulty learning tasks. This cluster of nodes suggests key relationships between physical impairment and completion of daily tasks, in which it is likely that, unsurprisingly, physical injury or limitations contribute to difficulty completing day to day tasks and responsibilities. This may exacerbate strain and stress within a family unit, compounding the effects of prior PTEs. Indeed, research on refugee populations documents the comorbidity between physical disability and psychological distress [26].

Lastly, this work provides an opportunity to deepen previous emic approaches with these data. The archival data were collected using culturally and locally adapted measures of stressors and distress. Using dynamic networks allowed us to identify connections between problems that were specific to the population and setting. Whereas latent and composite variables are useful for producing generalizable findings, dynamic network results provide more specificity that can facilitate setting-specific responses.

### Limitations

In contrast to some arguments about dynamic networks, we do not believe that concentration networks provide adequate evidence of causality between network elements (such as might be inferred from longitudinal or experimental data). Partial correlations are indeed useful for positing potential causal connections, but they may also indicate doublets—i.e., responses that are associated because their stimuli are essentially the same (or very similar). Measuring the same phenomenon using slightly different terminology is not at all uncommon in modern measurement, resulting in strong associations between responses, but probably does not result in actionable information for those designing interventions. Still, formative researchers might elect to combine composite measures with dynamic networks, yielding broader conceptualization of overarching sets of problems alongside network visualizations of the links between individual problems, as illustrated in this paper. Some may argue that the technical expertise needed to run dynamic network analyses outweighs their utility. However, although technically complex, networks are conceptually simpler than latent variables, and their specificity addresses some of the problems with composite variables. Technical expertise in dynamic networks in psychology and psychiatry is a growing area that is ripe for further implementation by applied researchers.

## Conclusions

In the current study we have applied a dynamic networks approach to a psychosocial dataset in a refugee population. We demonstrated findings that were more specific and actionable than previous latent and composite variable analyses; we argue that dynamic network approaches are of particular use to formative researchers, who are interested in identifying the particular needs of specific populations and maximizing limited resources. Useful psychosocial needs assessment should be specific, and we believe that measuring and mapping dynamic networks of stressors and symptoms allows for such specificity.

## Additional files

Additional file 1: Variable Name Abbreviations Used in Network Visualizations*.* This table is included to aid interpretation of the network visualizations in this paper. It identifies node abbreviation descriptions of all nodes in the network visualization. (DOCX 103 kb)

Additional file 2: Results of Network Edge Accuracy Analyses. This figure displays the results of analyses pertaining to network accuracy. To evaluate network edge accuracy, bootstrapping techniques are used to calculate the 95% confidence intervals of network edges. This figure depicts the bootstrapped 95% confidence intervals for estimated GLASSO network edge values (DOCX 95 kb)

Additional file 3: Results of Network Stability Analyses. This figure displays the results of analyses pertaining to network stability. To evaluate network stability, we compared centrality values from the original sample to values from increasingly smaller bootstrapped subsamples, yielding the greatest proportion of cases that may be dropped from the sample while maintaining a correlation of *r* greater than or equal to 0.70 between original and bootstrapped values. This figure depicts the average correlations between original centrality values of the GLASSO network and centrality values after dropping increasing percentages of participants from the sample. (DOCX 124 kb)

## Data Availability

The datasets used for the current study are available from the corresponding author upon reasonable request.
