# Supplementary material for: Formative psychosocial evaluation using dynamic networks: trauma, stressors, and distress among Darfur refugees living in Chad
Source: Confl Health. 2019 Jun 26;13:30. doi: 10.1186/s13031-019-0212-2 (PMC6595582; doi:10.1186/s13031-019-0212-2)
Supplement: Supplementary file 1 — Variable Name Abbreviations Used in Network Visualizations. This table is included to aid interpretation of the network visualizations in this paper. It identifies node abbreviation descriptions of all nodes in the network visualization. (DOCX 103 kb) [file 13031_2019_212_MOESM1_ESM.docx]

*Variable Name Abbreviations Used in Network Visualizations*

| Abbrev-iation | Variable Description | Abbrev-iation | Variable Description |
| --- | --- | --- | --- |
| *PTEs* |  | *Impairment* |  |
| Ampu | Having limb amputation | Act | Joining community activities |
| Beat | Being beaten | Conc | Concentrating on something |
| Bomb | Being bombed | Drs | Getting dressed |
| Bound | Being bound | Emo | Emotionally affected by health |
| Burn | Being burnt | Frd | Maintaining a friendship |
| Captv | Being held captive | Imp | Impact on life |
| Chase | Being chased | Resp | Managing household responsibilities |
| Cut | Being stabbed or cut | Stnd | Standing for long periods |
| Drown | Being drowned | Str | Dealing with strangers |
| Kidnp | Being kidnapped | Task | Learning a new task |
| Sexvl | Sexual violence | Walk | Walking a long distance |
| Shot | Being shot | Wash | Washing whole body |
| Suff | Being suffocated or strangled | Work | Day to day work |
| *Displacement Stressors (Basic Needs)* | | *Hozun* |  |
| Food | Getting food | Anger | Irritability or outburst of anger |
| Latr | Accessing latrines | App | Lack of appetite |
| Med | Getting medical help | Cry | Crying uncontrollably |
| Prvcy | Finding privacy | Flash | Flashbacks |
| Shelt | Finding shelter | Forget | Forgetfulness |
| Water | Getter water | Guilt | Feeling bad about surviving |
| Wood | Getting firewood | Head | Headaches |
|  | | Hpls | Feeling hopeless about the future |
| *Displacement Stressors (Safety)* | | Lonely | Feeling lonely |
|  |  | Mel | Feeling melancholy |
| Guard | Problems with camp guards | Night | Recurrent nightmares |
| Local | Problems with locals near camp | Palp | Palpitations |
| Mili | Threats from militant groups | React | Physiological reactivity at cues |
| Rebel | Recruitment by rebels in camp | Sad | Deep sadness |
| Res | Problems with camp residents | Sleep | Difficulty falling asleep |
| Safe | Not feeling safe in camp | Thghts | Recurrent thoughts |
| Sxlvl | Sexual assault in or near camp | Think | Thinking too much |
| Theft | Property taken by others | Torm | Being tormented |
