# Supplementary material for: Formative psychosocial evaluation using dynamic networks: trauma, stressors, and distress among Darfur refugees living in Chad
Source: Confl Health. 2019 Jun 26;13:30. doi: 10.1186/s13031-019-0212-2 (PMC6595582; doi:10.1186/s13031-019-0212-2)
Supplement: Supplementary file 2 — Results of Network Edge Accuracy Analyses. This figure displays the results of analyses pertaining to network accuracy. To evaluate network edge accuracy, bootstrapping techniques are used to calculate the 95% confidence intervals of network edges. This figure depicts the bootstrapped 95% confidence intervals for estimated GLASSO network edge values (DOCX 95 kb) [file 13031_2019_212_MOESM2_ESM.docx]

*Results of Network Edge Accuracy Analyses*


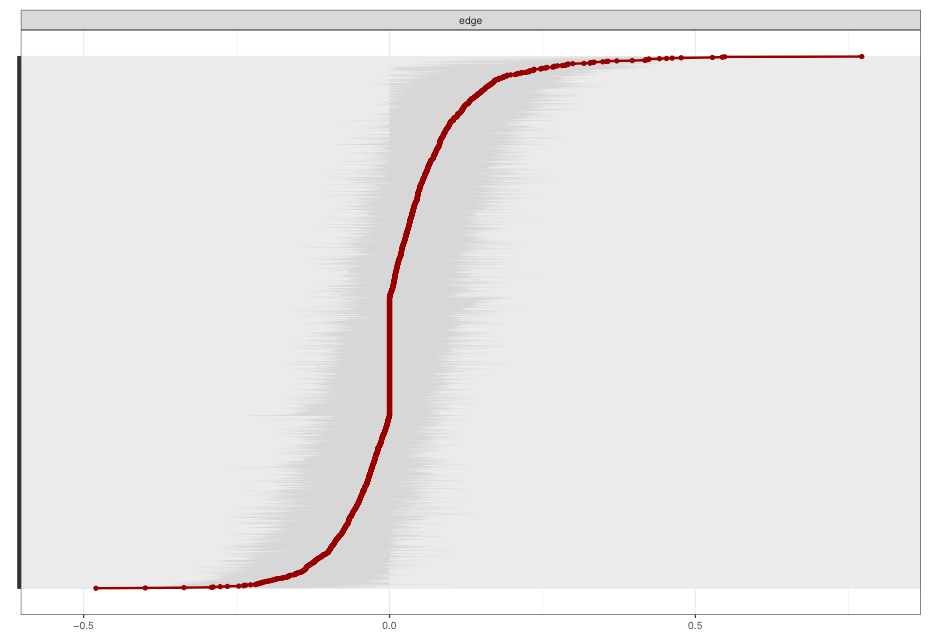


*Note*. Bootstrapped 95% confidence intervals for estimated GLASSO network edge values. The red line represents edge values, and the gray regions indicate confidence intervals.
