# Supplementary material for: Formative psychosocial evaluation using dynamic networks: trauma, stressors, and distress among Darfur refugees living in Chad
Source: Confl Health. 2019 Jun 26;13:30. doi: 10.1186/s13031-019-0212-2 (PMC6595582; doi:10.1186/s13031-019-0212-2)
Supplement: Supplementary file 3 — Results of Network Stability Analyses. This figure displays the results of analyses pertaining to network stability. To evaluate network stability, we compared centrality values from the original sample to values from increasingly smaller bootstrapped subsamples, yielding the greatest proportion of cases that may be dropped from the sample while maintaining a correlation of r greater than or equal to 0.70 between original and bootstrapped values. This figure depicts the average correlations between original centrality values of the GLASSO network and centrality values after dropping increasing percentages of participants from the sample. (DOCX 124 kb) [file 13031_2019_212_MOESM3_ESM.docx]

*Results of Network Stability Analyses*


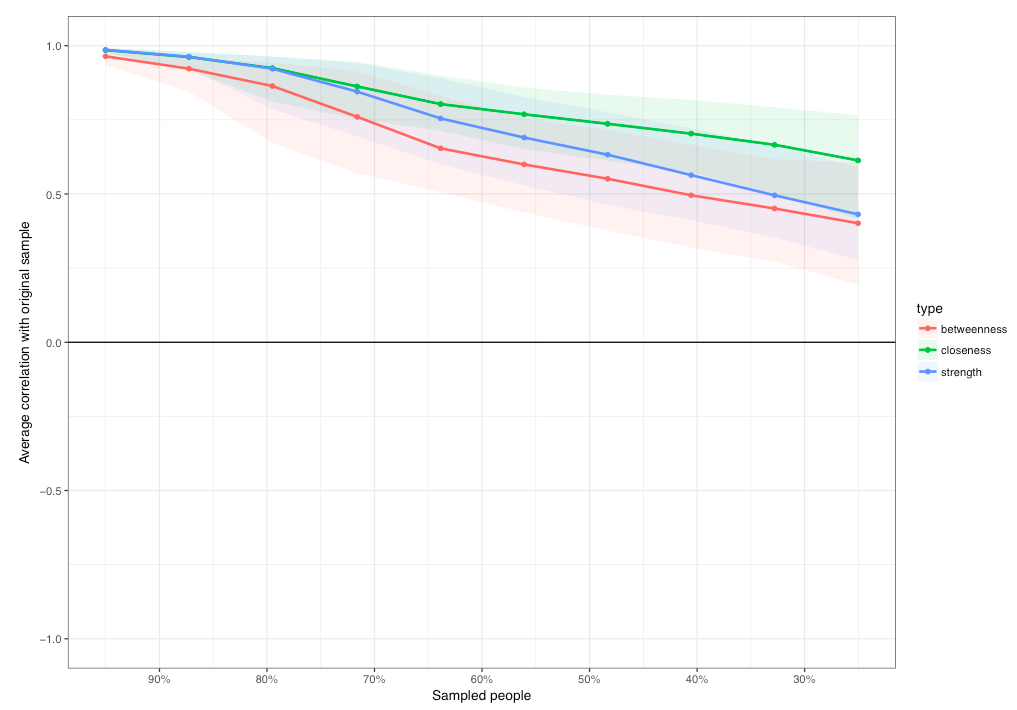


*Note.* Average correlations between original centrality values of the GLASSO network and centrality values after dropping increasing percentages of participants from the sample.
